# Performance of fracture risk assessment tool in HIV‐positive male individuals aged ≥45 years on suppressive antiretroviral therapy

**DOI:** 10.1002/jia2.25383

**Published:** 2019-08-18

**Authors:** Mao‐Song Tsai, Jun‐Yu Zhang, Hsin‐Yun Sun, Wen‐Chun Liu, Pei‐Ying Wu, Chia‐Jui Yang, Chien‐Ching Hung

**Affiliations:** ^1^ Department of Internal Medicine Far Eastern Memorial Hospital New Taipei City Taiwan; ^2^ School of Medicine College of Medicine Fu Jen Catholic University New Taipei City Taiwan; ^3^ Center of Infection Control National Taiwan University Hospital Taipei Taiwan; ^4^ Department of Internal Medicine National Taiwan University Hospital and National Taiwan University College of Medicine Taipei Taiwan; ^5^ School of Medicine National Yang‐Ming University Taipei Taiwan; ^6^ Department of Tropical Medicine and Parasitology National Taiwan University College of Medicine Taipei Taiwan

**Keywords:** bone mineral density, osteoporosis, osteopenia, combination antiretroviral therapy, ageing

## Abstract

**Introduction:**

An age‐specific evaluation and management algorithm for reduced bone mineral density (BMD) is suggested for HIV‐positive patients without major risk factors. Whether combination of BMD and the Fracture Risk Assessment Tool (FRAX) may detect more individuals for therapeutic interventions remains unclear. We aimed to determine the prevalence of middle‐aged or older HIV‐positive males fitting the criteria of therapeutic interventions with different approaches.

**Methods:**

From July 2016 to February 2018, HIV‐positive male patients aged ≥45 years receiving suppressive antiretroviral therapy were recruited in a cross‐sectional study, at two designated hospitals for HIV care in northern Taiwan. Patients with malignancy, AIDS, pre‐existing bone disease or immobilization were excluded. Information on clinical and demographic characteristics, FRAX questionnaire, activity questionnaire, BMD and serum 25(OH)D was obtained. FRAX scores combined with BMD (FRAX/BMD) and without BMD (FRAX) were calculated. The data were analysed on the basis of major risk factors for fragility fracture and age stratification, FRAX score and BMD results respectively.

**Results:**

We enrolled 330 patients with a mean age of 51.6 years and CD4 610 cells/μL, in whom 98.1% (n = 324) underwent BMD assessment of one site or more. By FRAX, 6.7% (n = 22) reached treatment thresholds (10‐year risk of major osteoporotic fracture ≥20% and/or hip fracture ≥3%). The prevalence of osteopenia (−2.5 <T‐score <−1) and osteoporosis (T‐score ≤−2.5) was 50.3% and 10.8% respectively. Compared with FRAX, FRAX/BMD identified 17.4% (95% CI 12.0% to 22.8%) more individuals who reached treatment thresholds (24.1% vs. 6.7%); even in the low‐risk group (without major risks for fragility fracture, 45 to 49 years, n = 129), FRAX/BMD identified 12.6% (95% CI 7.9% to 19.7%) more candidates (12.6% vs. 0%). Patients with BMI<22 kg/m^2^ (adjusted OR (aOR) 2.86, 95% CI 1.62 to 5.05) and aged ≥50 years (aOR 3.57, 95% CI 1.92 to 6.66) were more likely not to be identified as requiring treatment by FRAX but would be identified as requiring treatment by FRAX/BMD. The sensitivity and specificity of FRAX to detect candidates for interventions was 18.2% (95% CI 10.3% to 28.6%) and 97.9% (95% CI 95.2% to 99.3%) respectively.

**Conclusions:**

With FRAX as a screening approach among HIV‐positive male patients aged ≥45 years, addition of BMD assessment may detect more candidates for therapeutic management.

## 1 Introduction

Prevention of fragility fracture is one of the paramount issues for HIV‐positive individuals with improved survival 1. Osteoporotic fractures, mainly at the hip, vertebrae and distal forearm, are associated with significant morbidity, mortality and reduced quality of life, which, along with a high prevalence of comorbidities, may contribute to a significant economic burden in the long‐term successful management of HIV infection 2, 3, 4, 5, 6.

The incidence of fractures in HIV‐positive patients was higher than that in HIV‐negative individuals. Shiau *et al*. in a meta‐analysis found a crude incidence ratio of 1.58 (95% confidence interval (CI) 1.25 to 2.00) for any fracture in HIV‐positive patients compared with HIV‐negative individuals 7. Similarly, another meta‐analysis including 10 studies showed that HIV‐positive patients had a 2.17‐fold greater risk for all fractures than HIV‐negative individuals 1. It is important to note that 19 out of the 20 studies included in these two meta‐analyses were carried out in men aged 36 to 55 years 1, 7.

In 2006, a meta‐analysis of cross‐sectional studies reported that the respective rate of osteopenia and osteoporosis was 67% and 15% in HIV‐positive patients, with a 6.4‐ and 3.7‐fold higher odds for osteopenia and osteoporosis, respectively, compared with HIV‐negative controls 8. Moreover, Bonjoch *et al*. found a marked progression towards bone demineralization in 28.1% (normal to osteopenia in 12.5% and osteopenia to osteoporosis in 15.6%) of 391 HIV‐positive patients with at least two dual‐energy X‐ray absorptiometry (DXA) scans at a median follow‐up interval of 2.5 years 9. These studies suggest that the identification of HIV‐positive individuals with a low bone mass can be useful for early linkage to prevention and intervention of osteoporosis.

The WHO developed web‐based fracture risk assessment tool, FRAX, to calculate the 10‐year probability of both a hip fracture and a major osteoporotic fracture (https://www.sheffield.ac.uk/FRAX/). The European AIDS Clinical Society (EACS) and the Osteo Renal Exchange Program (OREP) recommend the FRAX algorithm being used for all HIV‐positive individuals over 40 years 10, 11. FRAX alone is used as an initial screening method for HIV‐positive patients aged between 40 to 50 years without other fracture risk 10, 11, whereas FRAX incorporating clinical data and BMD (FRAX/BMD) is suggested for adults with major fragility fracture risk factors, postmenopausal women and men aged ≥50 years 10, 11. FRAX also substitutes for FRAX/BMD if the diagnostic resources are not easily obtained 10, 11. However, it remains uncertain whether universal DXA screening among the middle‐aged HIV‐positive patients who are receiving suppressive combination antiretroviral therapy (cART) will improve case‐finding compared to current recommendations by OREP. The aim of this cross‐sectional study was to evaluate if DXA scan in combination with FRAX could detect more candidates for pharmacologic interventions than FRAX alone in HIV‐positive males aged ≥45 years who received suppressive cART.

## 2 Methods

### 2.1 Study setting and population

From July 2016 to February 2018, HIV‐positive male patients aged ≥45 years who received cART with viral suppression (plasma HIV RNA load (PVL) <200 copies/mL) were invited to participate in this cross‐sectional survey at the National Taiwan University Hospital (NTUH) and Far Eastern Memorial Hospital (FEMH) in northern Taiwan. The exclusion criteria were malignancy, AIDS status, pre‐existing bone disease or immobilization and receipt of growth hormone, testosterone, bisphosphonates or chemotherapy. A standardized questionnaire was used to obtain information on medications, comorbidities and other risk factors for osteoporosis, and self‐reported physical activity. This study was approved by the Research Ethics Committee of NTUH (registration number, 201603036RIPB) and FEMH (registration number, 106,015‐F). The written informed consent was obtained from each study participant.

### 2.2 FRAX score

FRAX was assessed in all participants with the use of FRAX questionnaire to obtain information on age, race, sex, history of previous fractures, family history of hip fracture in one parent, glucocorticoid use (equivalent to ≥5 mg of prednisolone for ≥3 months), current smoking, rheumatoid arthritis, risk for secondary osteoporosis (history of type 1 diabetes mellitus, osteogenesis imperfecta, long‐standing untreated hyperthyroidism, hypogonadism or menopause at <45 years of age, chronic liver disease, long‐standing malnutrition, malabsorption) and alcohol intake (≥3 units/day). The estimated 10‐year probability of major osteoporotic and hip fracture was calculated for each subject with the use of the FRAX website calculator with country specific algorithm for Taiwan (http://www.shef.ac.uk/FRAX/index.aspx). The FRAX score results with and without femoral neck (FN) BMD were abbreviated to FRAX/BMD and FRAX respectively. FRAX‐HIV is defined when HIV is included as a cause of secondary osteoporosis 8, 12.

### 2.3 Risk stratification and OREP recommendations

The participants were categorized into high‐, moderate‐ and low‐risk groups.

The participants with major risk factors for fragility fracture, including (1) a previous history of fragility fracture or (2) glucocorticoid treatment for >3 months (≥5 mg of prednisone daily or equivalent) were high‐risk group urged to assess BMD 10. Moderate‐risk group included men ≥50 years of age who are suggested to be evaluated with DXA if available 10, whereas low‐risk group included the men aged 45 to 49 years without major fracture risk factors, who are not advised to be evaluated with DXA initially 10.

### 2.4 Physical activity

Physical activity (PA) was assessed using the International Physical Activity Questionnaire‐Short Form (IPAQ‐SF), Taiwan version, to record the time that participants spent being physically active within the prior seven days 12. A total weekly energy expenditure was calculated by multiplying the time spent in different activities by an average metabolic equivalent tasks (MET), expressed in MET‐minutes/week. Responses on the IPAQ‐SF were stratified into three categories defined as follows: low‐PA, individuals who did not meet criteria for moderate or high categories; moderate‐PA, individuals achieving a minimum of at least 600 MET‐minutes/week and high‐PA, individuals achieving a minimum of at least 3000 MET‐minutes/week.

### 2.5 Laboratory investigations

Routine laboratory testing was performed every three to six months by following the national HIV treatment guidelines in Taiwan. PVL was quantified using the CobasAmplicor HIV‐1 Monitor test (CobasAmplicor version 1.5, Roche Diagnostics Corporation, IN) with a lower detection limit of 20 copies/mL, and CD4 lymphocyte count was determined using FACFlow (BD FACS Calibur, Becton Dickinson, CA). Total 25‐hydroxy‐vitamin D (Vit 25(OH)D) was measured using a commercially available radioimmunoassay (DiaSorin, Stillwater, MN, USA) within three months of sample collection.

### 2.6 BMD assessment

The BMD and T‐score of the lumbar spine (LS) (L1‐L4), total hip (TH) and FN were measured using the same DXA device (Lunar Prodigy; GE Healthcare, Belgium). Based on the WHO criteria, osteopenia is defined as a BMD T‐score between −1.0 and −2.5, and osteoporosis is defined as a BMD T‐score less than or equal to −2.5 13.

### 2.7 Thresholds for pharmacologic treatment

We compared the FRAX, FRAX‐HIV and FRAX/BMD for fracture prediction to identify the participants who might meet one of the pharmacologic treatment criteria based on the Taiwan osteoporosis guidelines and OREP recommendations, including T‐score ≤−2.5 at the LS, TH or FN, 10‐year probability of a hip fracture ≥3%, and 10‐year probability of a major osteoporosis‐related fracture ≥20% 10, 14.

### 2.8 Statistical analysis

Parametric data were compared using an analysis of variance (ANOVA), whereas nonparametric data, expressed as median and interquartile range (IQR), were compared using the Kruskal–Wallis test. Categorical variables were compared using the chi‐square test or the Fisher's exact test. Multivariate logistic regression model were applied to correlate the effect of the independent variables with the results of FRAX and FRAX/BMD. The confidence interval (CI) was set at 95%. All statistical tests were two‐tailed, and *p *<* *0.05 were considered to be statistically significant. The analysis was conducted using the statistical package SAS 9.4 (SAS Institute Inc., Cary, North Carolina, USA).

## 3 Results

### 3.1 Baseline characteristics

**Figure 1:**
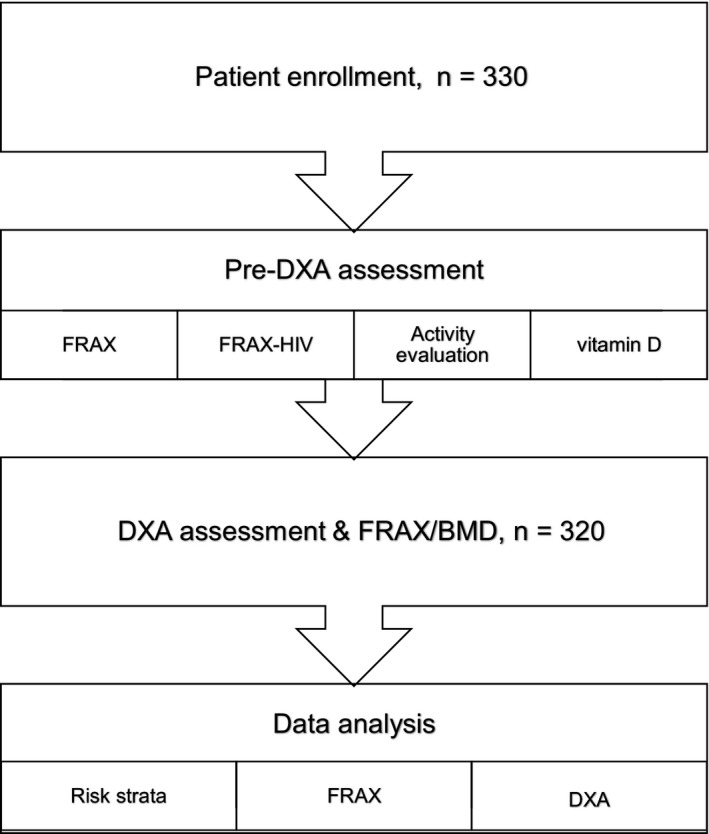
Study flow diagram.

The characteristics of the 330 participants are shown in Table 1, and the study flow diagram is shown in Figure 1. Our participants were mostly MSM (n = 254, 77.0%) with a mean age of 52.6 years (SD 7.5), and all participants were receiving suppressive cART with a mean CD4 count of 610 cells/μL and 90.9% having achieved PVL <20 copies/mL. Tenofovir disoproxil fumarate (TDF) was used in 92.4% of the participants and ritonavir‐boosted protease inhibitors (PIs) in 47.0%. Seventy‐one participants (21.5%) were classified as high‐risk group (glucocorticoid use, 9; and a history of fragility fracture, 62); 130 men (39.4%) aged 50 years or more as moderate‐risk group and 129 men (39.1%) aged 45 to 49 years as low‐risk group.

**Table 1:** Baseline characteristics of HIV‐positive participants ≥45 years by risk status

	Total, N = 330	High‐risk, N = 71	Moderate‐risk, N = 130	Low‐risk, N = 129	*p‐*value
Age, mean (SD), years	52.6 (7.5)	53.4 (7.5)	57.6 (7.2)	47.0 (1.7)	<0.01
45 to 49, n (%)^a^	158 (47.9)	29 (40.9)	0	129 (100)	
50 to 60	121 (36.7)	26 (36.6)	95 (73.1)	0	
>60	51 (15.5)	16 (22.5)	35 (26.9)	0	
Major risk factors					
Glucocorticoid use, n (%)	9 (2.7)	9 (12.6)	0	0	
Fragile fracture, n (%)	62 (18.8)	62 (87.3)	0	0	
Alcohol use, n (%)	21 (6.4)	6 (8.5)	7 (5.4)	8 (6.2)	0.69
BMI, mean (SD), kg/m²^b^	23.6 (3.4)	23.7 (3.8)	23.5 (3.3)	23.6 (3.2)	0.88
HIV transmission risk group, n (%)					
Heterosexuals	66 (20.0)	19 (26.8)	35 (26.9)	12 (9.3)	<0.01
MSM	254 (77.0)	48 (67.6)	91 (70.0)	115 (89.2)	
Others	10 (3.0)	4 (5.6)	4 (3.1)	2 (1.6)	
CD4 count, mean (SD), cells/μl	610 (255)	593 (233)	587 (275)	643 (243)	0.17
Plasma HIV‐1 RNA load <20 copies/mL, n (%)	300 (90.9)	71 (94.4)	119 (91.5)	114 (88.4)	0.35
ART					
TDF, n (%)	305 (92.4)	66 (93.0)	120 (92.3)	119 (92.3)	0.98
PI, n (%)	155 (47.0)	41 (57.8)	61 (46.9)	53 (41.1)	0.08

Data are presented as mean (SD) or n (%). ART, antiretroviral therapy; BMI, body‐mass index; MSM, men who have sex with men; NRTI, nucleoside/nucleotide reverse‐transcriptase inhibitor; PI, protease inhibitor; SD, standard deviation; TDF, tenofovir disoproxil fumarate.

^a^Percentages may not sum to 100 because of rounding; ^b^BMI is the weight in kilogrammes divided by the square if the height in metres.

Three‐hundred and eight participants (93.3%) completed PA questionnaire interview, with 28.3% (n = 87) categorized as having high PA (>3000 MET‐minutes/week), 41.9% (129) moderate (600 to 3000) and 29.9% (92) low (<600). The level of PA did not significantly differ among the high‐risk, moderate‐risk and low‐risk groups: 23.9%, 26.2% and 32.8%, respectively, with high PA; 50.7%, 44.3% and 34.5%, respectively, with moderate PA; and 25.4%, 29.5% and 32.8%, respectively, with low PA (*p *=* *0.26).

Two‐hundred and seven participants (62.7%) had plasma concentrations of Vit 25(OH)D level >20 ng/mL considered to be sufficient according to the Institute of Medicine recommendations, 29.4% considered to be insufficient (12 to 20 ng/mL), and 7.9% considered to be deficient (<12 ng/mL). There was no statistically significance among the high‐risk, moderate‐risk and low‐risk groups in terms of the category of 25(OH)D level (sufficient: 63.4%, 71.5% and 53.5%; insufficiency: 26.8%, 21.5% and 38.8%; and deficiency: 9.9%, 6.9% and 7.8% respectively) (*p *=* *0.23).

### 3.2 Ten‐year estimated fracture risks by FRAX and FRAX‐HIV

The mean estimated 10‐year risk of fracture by FRAX alone at major osteoporosis sites was 3.7%, and the estimated risk was significantly higher in the high‐risk group (6.5%) than moderate‐risk (3.9%) and low‐risk (2.0%) groups (*p *<* *0.01). One (0.3%) patient had 10‐year major osteoporosis‐related fracture risk ≥20% by FRAX (Table 2). The mean estimated 10‐year risk of fracture by FRAX at the hip was 0.9%, which was significantly higher in the high‐risk group (high‐risk 2.0%, moderate‐risk 1.1% and low‐risk 0.2%, *p *<* *0.01) (Table 2). Twenty‐two participants (6.7%) had a hip fracture risk ≥3% based on the FRAX results.

**Table 2:** Ten‐year estimated fracture at major osteoporosis and the hip: FRAX, FRAX‐HIV and FRAX/BMD by study groups

	Total	High‐risk	Moderate‐risk	Low‐risk
FRAX	N = 330	N = 71	N = 130	N = 129
Major osteoporotic fracture, mean (SD), %	3.7 (2.8)	6.5 (3.8)	3.9 (2.2)	2.0 (0.6)
10‐year major osteoporosis‐related fracture ≥20%, n (%)	1 (0.3)	1 (1.4)	0	0
Hip fracture, mean (SD), %	0.9 (1.8)	2.0 (3.0)	1.1 (1.6)	0.2 (0.1)
10‐year hip fracture ≥3%, n (%)	22 (6.7)	11 (15.5)	11 (8.5)	0
FRAX‐HIV	N = 330	N = 71	N = 130	N = 129
Major osteoporotic fracture, mean (SD), %	5.2 (4.1)	9.1 (5.4)	5.5 (3.3)	2.7 (0.8)
10‐year major osteoporosis‐related fracture ≥20%, n (%)	4 (1.2)	3 (4.2)	1 (0.8)	0
Hip fracture, mean (SD), %	1.6 (3.0)	3.3 (4.7)	1.8 (2.8)	0.3 (0.1)
10‐year hip fracture ≥3%, n (%)	44 (13.3)	26 (36.6)	18 (13.8)	0
FRAX/BMD	N = 320	N = 67	N = 127	N = 126
Major osteoporotic fracture, mean (SD)%	5.6 (4.8)	9.3 (6.8)	5.7 (3.7)	3.6 (2.9)
10‐year major osteoporosis‐related fracture ≥20%, n (%)	10 (3.1)	7 (10.4)	2 (1.6)	1 (0.8)
Hip fracture, mean (SD), % z	2.3 (3.6)	4.1 (5.4)	2.3 (3.0)	1.4 (2.5)
10‐year hip fracture ≥3%, n (%)	73 (22.8)	26 (38.8)	35 (27.6)	12 (9.5)

BMD, bone mineral density; FRAX, Fracture Risk Assessment Tool; SD, standard deviation.

Based on FRAX‐HIV, four participants (1.2%) had major osteoporosis‐related fracture risk ≥20%, and 44 (13.1%) had a hip fracture risk ≥3%. No participants in the low‐risk group were found to have major osteoporotic fracture risk ≥10% or hip fracture risk ≥3%.

### 3.3 BMD and osteoporosis

Six participants (1.7%) declined the BMD assessment. The BMD data and the distributions of osteoporosis at the LS, TH and FN are shown in Table 3. The overall prevalence of osteoporosis was 10.8% (35/324; 95% CI 7.8% to 14.5%). Osteoporosis was 15.9%, 6.3% and 12.6% for the high‐, moderate‐ and low‐risk group respectively (*p *=* *0.08). The prevalence of osteoporosis did not significantly differ in the low‐risk group versus the moderate‐ and high‐risk groups (difference 3.0%, 95% CI −4.3% to 10.1%). Of note, we found a sensitivity of 40.5% (95% CI 25.6% to 56.7%) in detecting osteoporosis at the LS, which was significantly lower than the performance at the TH (71.4%, 95% CI 53.7% to 85.4%) or at FN (62.9%, 95% CI 44.9% to 78.5%). The participants with osteoporosis had a significantly lower BMI than those without (20.8 kg/m^2^ (95% CI 20.0 to 21.7) vs. 23.9 kg/m^2^ (95% CI 23.5 to 24.3)), and no significant difference in terms of age distribution, Vit 25(OH)D level, TDF use or PI use was found.

**Table 3:** BMD at the lumbar spine (LS), total hip (TH) and femoral neck (FN) by study groups

BMD	Total	High‐risk	moderate‐Risk	Low‐risk	*p‐*value
LS BMD‐g/cm^2^, mean (SD)	N = 323 1.075 (0.164)	N = 68 1.059 (0.161)	N = 128 1.083 (0.171)	N = 127 1.075 (0.158)	0.62
LS Mean T‐score	−0.38	−0.56	−0.27	−0.40	
LS T ≤−2.5, n (%)	13 (4.0)	4 (5.9)	3 (2.3)	6 (4.7)	0.43
TH BMD‐g/cm^2^, mean (SD)	N = 320 0.899 (0.134)	N = 67 0.881 (0.155)	N = 127 0.907 (0.125)	N = 126 0.902 (0.134)	0.47
TH Mean T‐score	−0.49	−0.64	−0.42	−0.48	
TH T ≤−2.5, n (%)	26 (1.9)	10 (14.9)	7 (5.5)	9 (7.0)	0.06
FN BMD‐g/cm^2^, mean (SD)	N = 32 0.836 (0.130)	N = 67 0.827 (0.140)	N = 127 0.833 (0.128)	N = 126 0.845 (0.126)	0.61
FN Mean T‐score	−0.97	−1.06	−0.97	−0.93	
FN T ≤−2.5, n (%)	27 (8.4)	9 (13.4)	5 (3.9)	13 (10.3)	0.05
Any sites T ≤−2.5, n (%)	35 (10.8)	11 (15.9)	8 (6.3)	16 (12.6)	0.08

BMD, bone mineral density; FN, femoral neck; TH, total hip; LS, lumbar spine; SD, standard deviation.

### 3.4 Frax/bmd

The mean estimated 10‐year risk of fracture by FRAX/BMD was 5.6% at major osteoporosis sites (*p *<* *0.01) and 2.3% at the hip (Table 2). The major osteoporotic fracture by FRAX/BMD was 9.3% for the high‐risk group, 5.7% the moderate‐risk and 3.6% for the low‐risk group (*p *<* *0.01). A similar trend was observed for the risk of hip fracture by FRAX/BMD: 4.1% for the high‐risk, 2.3% moderate‐risk and 1.4% low‐risk group (*p *<* *0.01). Based on FRAX/BMD, 3.1% had major osteoporosis‐related fracture risk ≥20% and 22.8% had a hip fracture risk ≥3%.

### 3.5 Candidates for pharmacologic treatment using the three screening approaches

**Figure 2:**
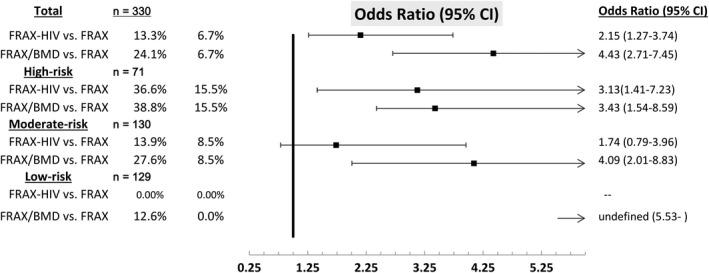
Distributions of FRAX, FRAX‐HIV and FRAX/BMD achieving thresholds for pharmacologic treatment by study groups.

By FRAX/BMD, 24.1% (77/320) met the thresholds for pharmacologic treatment endorsed by the guidelines, which was significantly higher than that by FRAX alone (6.7%) (OR 4.42, 95% CI 2.71 to 7.45) (Figure 2) or by FRAX‐HIV (13.3%) (OR 1.81, 95% CI 1.29 to 2.53) 10, 14. Of the 77 candidates meeting the thresholds for pharmacologic treatments by FRAX/BMD, 42.9% (n = 33) had T‐score ≤−2.5, 55.8% (43) had −2.5 <T‐score <−1 and 1.3% (1) had normal bone density. The FRAX and FRAX/BMD produced identical fracture risk predictions for 79.7% of the participants. BMI <22 kg/m^2^ (adjusted OR (aOR) 2.86, 95% CI 1.62 to 5.05) and aged ≥50 years (aOR 3.57, 95% CI 1.92 to 6.66) were the associated factors with fracture risk for different predictions.

The sensitivity and specificity of screening algorithm based on risk stratification was 79.2% (95% CI 68.5% to 87.6%) and 100.0% (95% CI 98.6% to 100%) respectively. The subgroup analysis showed that more candidates for pharmacologic treatment would be identified by FRAX/BMD than FRAX in the high‐risk (38.8% vs. 15.5%, *p *<* *0.01), moderate‐risk (27.6% vs. 8.5%, *p *<* *0.01) and low‐risk groups (12.6% vs. 0%, *p *<* *0.01) (Figure 2). Among the high‐risk participants, BMI <22 kg/m^2^ (aOR 3.64, 95% CI 1.09 to 12.1) and low‐PA (aOR 7.34, 95% CI 2.05 to 26.2) were the factors associated with fracture risk for different predictions by FRAX and FRAX/BMD. BMI <22 kg/m^2^ (aOR 3.67, 95% CI 1.47 to 9.17) and aged ≥60 years (aOR 6.16, 95% CI 2.43 to 15.63) were the factors associated with fracture risk for different predictions. Among low‐risk participants, BMI <22 kg/m^2^ (aOR 8.59, 95% CI 2.30 to 32.1) was the only associated factor.

Using FRAX scores with cutoff values ≥10% for DXA assessment, only eight participants were eligible to be screened, with the sensitivity of 10.4% and specificity of 100%. While including HIV as a secondary cause of osteoporosis, FRAX‐HIV showed a significantly higher sensitivity of 27.3% (difference 16.9%, 95% CI 4.8% to 28.9%) and lower specificity of 96.3% (difference −3.7%, 95% CI −1.3 to −6.1%).

The DXA screening without estimating fracture risk had a sensitivity of 42.8% (95% CI 31.6% to 54.7%). All participants with osteoporosis (35/35) fitted the criteria for therapeutic interventions, whereas 25.6% of participants with osteopenia (41/160) and 0.8% of participants with normal BMD (1/129) fitted the criteria.

Medical records of the participants within one year after enrolment were retrospectively reviewed and two (0.6%) incident factures were identified. One 63‐year‐old male with a prior fracture history had an episode of right femoral fracture and the other 44‐year‐old male without risk factors had a fracture at the right proximal fibula who had low FRAX scores (1.5% major osteoporosis and 0.1% at the hip) but low BMD (T‐score −3.3 at the FN).

## 4 Discussion

In this study evaluating the performance of the algorithm developed for HIV‐positive individuals for screening, diagnosis and management of bone disease, we found that 12.6% of the males without clinical risk factors in the 45 to 49 years age strata (low‐risk) may miss the opportunity of therapeutic interventions to reverse bone loss and to alter the osteoporosis status. FRAX or DXA alone as alternative diagnosis or screening tool has high specificity, but the sensitivity may be a concern. Low BMI (<22 kg/m^2^) was one of the factors accounting for the different results between FRAX and FRAX/BMD. High‐risk individuals with low‐PA (<600 MET‐minutes/week) and moderate‐risk individuals aged >60 years could be the two prioritized groups for BMD assessment.

People with a T‐score ≤−2.5 may be at higher risk of fracture but they did not account for the majority of fracture cases 15, 16. The FRAX designed to allow for the calculation without the BMD data has been proposed as a screening tool with consideration of both treatment effectiveness and cost‐benefit issues. However, the studies in HIV‐positive individuals suggest that fracture estimates using FRAX were likely to underestimate the fracture risk 17, 18. Accuracy was improved if HIV was considered a cause of secondary osteoporosis in FRAX calculation, but still not good enough compared with FRAX/BMD. When available, DXA combined with FRAX may be a better screening modality to determine whether to start pharmacologic therapy. Our data revealed a statistically significant difference of risk estimation between FRAX and FRAX/BMD, +2.0% (95% CI 1.6 to 2.3) for major osteoporotic fractures and + 1.5% (95% CI 1.2 to 1.8) for hip fractures prediction, which implies limited precision using clinical risk factors alone in predicting osteoporosis severe enough to initiate treatment. Our results are consistent with the findings of underestimation of the fracture risk in HIV‐positive patients from two large cohorts, Veterans Aging Cohort Study (VACS) for men 19. Our study also demonstrated improvement of case‐finding with FRAX‐HIV (compared with FRAX, OR 2.15, 95% CI 1.27 to 3.74), which lends support for the recommendations to include HIV as a secondary cause of osteoporosis when utilizing the FRAX calculator 19, 20.

Net bone loss is a silent process at a rate of 0.3% to 0.5% per year in the midlife (aged 35 to 45 years) as part of ageing process 21. Ageing in combination with intrinsic and extrinsic factors such as HIV infection may accelerate bone demineralization and deterioration of bone micro‐architecture 22, 23. Our previous study found that reduced BMD (osteopenia 35.6% and osteoporosis 3.8%) was prevalent among HIV‐positive Taiwanese with a median age of 37 years 24. Recently, one French study found 15.6% of HIV‐positive young men (<50 years, median age 43 years) on suppressive cART had osteoporosis 25. In the multinational EuroSIDA study of 11,820 HIV‐positive patients, there were 619 incident fractures 26; of note, the median age of the patients with fractures was 50 years and 97% of them received cART 26. These studies highlight an important clinical issue in identifying individuals who may benefit from pharmacological interventions. Our analyses show that 12.6% of the low‐risk participants who had developed osteoporosis did not meet the treatment or warning thresholds (>10% major osteoporotic fracture) by either FRAX or FRAX‐HIV, which gives a practical demonstration of the complementary role of BMD for fracture risk assessment 10.

Despite the fact that cART initiation may further worsen, rather than ameliorate, BMD loss of 2% to 6% at the hip and the spine within the first 24 months similar to the decline within the initial phase of menopause, osteoporosis screening is often overlooked and viewed as a low priority 27, 28, 29, 30, 31, 32. We found that BMI was an important contributing factor in the occurrence of discrepancies between FRAX and FRAX/BMD. In our study, BMI of 22 kg/m^2^ can be considered a factor to determine participants’ priority in different risk groups. In high‐risk group, physical activity at 600 MET‐minutes/week was an alternative determinant and is one of potentially modifiable factors linked to BMD and fractures 33, 34. We could not find the roles of other potentially modifiable factors such as TDF (or PIs) exposure and serum Vit 25(OH)D level in our study.

To our knowledge, this is the first study in the Asia‐Pacific region to evaluate the role of DXA in the fracture risk assessment using the FRAX algorithm in HIV‐positive individuals over 40 years of age. More than 95% participants had LS, FN and TH BMD data, which improved the sensitivity of osteoporosis detection and minimized the bias from its discordance. This study also has limitations that warrant caution. First, being a cross‐sectional study precluded us from establishing the causal relationships and exploring associated factors with longitudinal follow‐up clinical events. The number of fracture remained low in our participants and long‐term follow‐up is warranted. Second, since many participants did not initiate HIV treatment at these two hospitals when the study was conducted, we could not find the association between bone loss and the individual antiretrovial agent and its exposure duration, such as TDF and PIs. Tenofovir alafenamide (TAF) and integrase inhibitors were recommended by most guidelines, and the future studies of TAF and integrase inhibitors in ageing HIV‐positive population are highly anticipated 35, 36, 37. Third, the sample size was relatively small, and there was no HIV‐negative comparator group. Fourth, all participants in our study were middle‐aged males and the results may not be generalizable to all HIV‐positive patients or females in Taiwan or other countries. Fifth, we did not have any information on the biochemical markers of bone remodelling and gonadal status of our participants. Last, one of the major risk factors for fragility fracture, high risk for falls, by OREP was not considered in our study. Fall history and fall prevention are imperative issues for fracture prevention. Yet, we cannot find a widely used metric for the assessment of fall risk.

## 5 Conclusions

We conclude that FRAX can be easily applied to HIV‐positive patients but may underestimate the risk of fracture. If available, DXA may be a better screening modality to determine whether to start pharmacologic therapy in HIV‐positive male patients aged ≥45 years, especially the individuals with risk factors.

## Competing interests

The funder (FEMH) had no role in study design, data collection or in analysis and interpretation of the results, and this paper does not necessarily reflect views or opinions of the funder. Hung CC has the following competing interests: research support from Janssen, Merck, Gilead Sciences and ViiV, speaker honoraria from Abbvie, Gilead Sciences and ViiV, and serving on advisory boards for Gilead Sciences and ViiV. While another one author, Yang CJ, has the following competing interests: speaker honoraria from Abbvie, Gilead Sciences and ViiV. Other authors have no competing interest to disclose.

## Authors’ contributions

Hung CC and Yang CJ were the chief investigators and designed the protocol. Tsai MS, Zhang JY, Sung HY, Liu WC and Wu PY coordinated the data collection and regulatory requirements. Tsai MS and Yang CJ conducted the statistical analyses and data interpretation. Tsai MS, Yang CJ and Hung CC performed the literature search and review and wrote the manuscript; Yang CJ and Hung CC edited and revised the manuscript. All authors approved the final version of the manuscript.
